# Topiramate intoxications & hemodialysis – Literature review and the first case report of a massive suicidal intoxication treated with hemodialysis

**DOI:** 10.1016/j.toxrep.2022.08.004

**Published:** 2022-08-12

**Authors:** Tim Schutte, Anne van Tellingen, Janneke van den Broek, Marloes ten Brink, Marleen G. van Agtmael-Boerrigter

**Affiliations:** a Amsterdam UMC location Vrije Universiteit Amsterdam, Department of Internal Medicine & Department of Medical Oncology, Boelelaan, Amsterdam 1117, the Netherlands; b Zaans Medisch Centrum, Zaandam, the Netherlands

**Keywords:** Topiramate intoxications, Dialysis, Hemodialysis

## Abstract

**Background:**

Topiramate is an anticonvulsant from sulfamate-substituted monosaccharides that is increasingly used to treat migraines. Serious topiramate intoxications have been described. Unfortunately, indications for and the effect of interventions, including hemodialysis, in severe intoxications seem expert-based and lack empirical evidence. We aim to review the literature on topiramate intoxication cases and to describe the first topiramate intoxication with toxicokinetic data following treatment with hemodialysis.

**Methods:**

A literature review was conducted using the PubMed database. Included articles were reviewed for symptoms; management, including acute hemodialysis; toxicokinetic data; and outcomes.

**Results:**

We found 61 hits in the PubMed database and checked 392 references in the snowball search; 22 were included for data extraction, reporting 29 cases. The majority of the patients were female (n = 23/29, 79%), ranging in age from 2 to 44 years (median 21). The ingested topiramate amount ranged from 175 to 40,000 mg (usual maintenance dose of 50 mg BID and a general maximum of 500 mg BID). Topiramate concentrations were reported in eight cases, ranging from 3.7 to 356.6 mg/L (for reference, the therapeutic range is 2–30 mg/L). Serious topiramate intoxications can result in seizures, coma, hemodynamic instability and severe metabolic acidosis. In no single case was hemodialysis used.

**Conclusion:**

Serious symptoms of topiramate intoxications exist, and hemodialysis is used infrequently. If symptoms are refractory to symptomatic treatment, hemodialysis can reduce topiramate concentrations and symptomatology.

## Highlights

• Topiramate intoxications are treated with supportive measures.
• Invasive treatments such as hemodialysis are infrequently used.
• Toxicokinetic data of topiramate intoxications are very limited.
• Hemodialysis enhances clearance of both topiramate and quetiapine.

## 1 Introduction

Topiramate is a commonly used drug and has multiple indications for its use, as an anti-epileptic, anti-migraine, mood-stabilizing and anti-depressant drug [1.], [2.], [3.]. Topiramate raises cerebral GABA levels, facilitates GABAergic neurotransmission and inhibits glutamatergic activity at AMPA or kainite receptors[4]. Moreover, topiramate is a carbonic anhydrase inhibitor, and, as a side effect, it can cause non-anion gap metabolic acidosis[4.], [5.]. Reported side effects of regular doses of topiramate include neuro(psychological) functions such as rigidity, paresthesia, effects on psychomotor functions and loss of appetite [4.], [5.], [6.], [7.], [8.], [9.]. When used in overdose, topiramate can cause varying degrees of sedation, coma, status epilepticus, hypotension, and severe metabolic acidosis, the latter presumably related to the carbonic anhydrase inhibition [10]. Despite these serious effects, previous case reports and series demonstrated overall mild effects and outcomes, even in severe intoxications with topiramate [11].

The risk of a severe intoxication with topiramate increases due to the growing number of users, from 14,909 users in 2011–20,286 users in 2019 in the Netherlands; this represents a 36% increase [12]. Given its indication as mood stabilizer, vulnerable patients use topiramate. Furthermore, previous case reports have described suicidal ideation following initiation of topiramate therapy [13.], [14.]. This could further enhance the risk of topiramate leading to suicide attempts.

In six months’ time, two patients attempted suicide with large amounts of topiramate and thereafter were seen in our emergency department. These cases were initially treated with supportive and symptomatic management, until symptoms became refractory in one case. In cases of refractory symptoms, acute hemodialysis can be considered, according to the Summary of Product Characteristics (SmPC) and the advice of the national toxicology information center [15]. Nevertheless, empirical evidence of the effectiveness of hemodialysis in topiramate intoxications seems to be lacking. Additionally, clearance of topiramate is both theoretically feasible given its pharmacokinetic properties and has been demonstrated in patients using regular doses of topiramate while on hemodialysis for end-stage renal disease [16.], [17.].

Now, we aim to summarize the literature regarding topiramate intoxications and its management and treatments, including hemodialysis, and outcomes. Moreover, we describe a case of a combined topiramate, venlafaxine and quetiapine intoxication with toxicokinetic data following hemodialysis.

## 2 Methods

### 2.1 General methodology

We reported a serious attempted suicidal intoxication with topiramate, venlafaxine and quetiapine. Additionally, we performed a systematic review on topiramate intoxications. The PRISMA guidelines for systematic reviews were followed where possible. Because of the diverse outcome measures and the nature of the case reports, no meta-analysis or additional analyses on risk of bias were performed.

### 2.2 Data sources and search strategy

We searched the PubMed database for cases of intoxications with topiramate using the following search criteria: (topiramate [Supplementary Concept] OR Topiramate [Title/Abstract]) AND (“suicide” [MeSH Terms] OR “drug overdose” [MeSH Terms] OR intoxication); moreover, all references of selected articles were screened and reviewed as stated in “study selection”. Full text articles were retrieved via the institutional library and/or via the corresponding authors.

### 2.3 Study selection

Our aim was to review individual reports of intoxications. Therefore, inclusion criteria were case reports of intoxications with topiramate (or poly-drug intoxications with topiramate) and case series with individual patient data of intoxications with topiramate (or poly-drug intoxications with topiramate) that were published in scientific journals written in English, German, French or Dutch. Exclusion criteria were reviews, meta-analysis or case series not reporting individual patient data and case reports of side effects of the use of regular doses of topiramate, preclinical studies or pharmacokinetic or pharmacodynamic studies with therapeutic or sub-therapeutic doses of topiramate. Although excluded, non-original research studies such as reviews, meta-analyses and the like were set aside to be used eventually for reference. All references of eligible articles were screened for eligibility in a snowball search based on the title and abstract, similar to the primary inclusion and exclusion criteria.

### 2.4 Data extraction

All eligible articles were assessed for study characteristics, including the year of publication, primary author, publication date and journal. All reported individual cases meeting the inclusion criteria were included.

Data were extracted using a coding sheet, and the coded or extracted items were patient characteristics, dose, relative dose to patient weight, regular or chronic topiramate usage, measured concentration, eventual co-ingestion, clinical symptoms, and treatment including dialysis and outcomes.

### 2.5 Data analysis

Extracted data were imported in SPSS (IBM, version 26). Descriptive statistics were used to report range and median for variables, given a presumed non-normal distribution.

## 3 Results

**Fig. 1:**
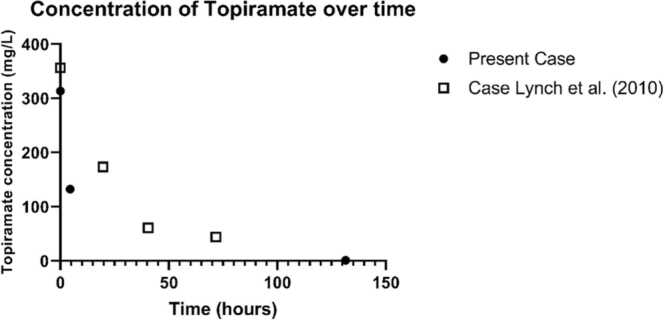
Concentration of topiramate over time of the present case with 3 h of hemodialysis in the first four hours (first two measurements of present case). As a reference case, we used the concentrations reported by Lynch et al., 2010 (no hemodialysis, female patient 37 years of age, unknown weight, 16:15 day 1, 356.6 μg/ml; 09:55 day 2, 173.6 μg/ml; 61.2 and 44.0 μg/ml on the third day of hospitalization at 0841 and 1555, respectively) [27]. T0 is time of presentation at the emergency department. Additional to the data in Table 2 (present case), a concentration of 1.1 mg/L was measured at Day 6. The reduction reported by Lynch et al. is 9.3 mg/L/hr (0-order kinetics). As a rough estimation, when this reduction is applied to our case, we would estimate a concentration of 313.5 mg/l – 4.5 hr * 9.3 mg/L/hr = 271.6 mg/L when no hemodialysis was used. The measured concentration of 132.5 mg/L, 4.5 h later after 3 h of dialysis suggests a considerable enhanced clearance, probably around 50% due to hemodialysis.

Case: A 41-year-old woman who intentionally ingested ~25,500 mg topiramate and other psychotropic drugs was admitted to the emergency room of a community or regional hospital in the Netherlands. She was hypotensive and comatose before suffering seizures that were resistant to conservative management. Within three hours we initiated hemodialysis and measured topiramate concentrations before (313.5 mg/L) and after three hours of hemodialysis (132.5 mg/L), with resolution of symptoms. See Table 1 for our complete case report. Moreover, drug concentrations pre- and post-dialysis of venlafaxine and quetiapine are provided (Table 2 and Fig. 1).

**Table 1:** Case report of a poly-drug intoxication with topiramate, venlafaxine and quetiapine. Report based on the ABCDE structure: A: airway; B: breathing; C: circulation; D: disabilities; E: environment.

A 41-year old women with a known depressive disorder was found unresponsive with a suicide letter. She was reported to have had rhythmic contractions of her arms and was transported to our emergency department. A: Obstructed airway due to low EMV (eye, motor, verbal response) score, patient is unresponsive. Anesthesiologist is called for intubation, and chin lift, mayo tube and balloon-mask ventilation were initiated. B: Breathing frequency 12/minute; symmetrical thorax excursions, at auscultation normal breading sounds. X-thorax showed no abnormalities. Arterial blood gas analysis revealed a non-anion gap metabolic acidosis. C: Hypotension with an RR 70/30 mmHg, capillary refill < 2 s, well circulated. D: E1 M4/5 V1; Pupils are equal and reactive to light (PEARL). E: Glucose 5.0 mmol/l; temperature 36 degrees Celsius. No neck stiffness, no apparent self-inflicted harm, weight 65 Kg, length 170 cm. She was last seen 6–7 h earlier and had hidden a large amount of empty pill strips which accounted for 510 tablets of topiramate 50 mg; 170 x venlafaxine 75 mg retard; 30 x quetiapine 25 mg and 8 x desloratadine 5 mg. Upon arrival we immediately started two peripheral venous catheters infusing Ringer’s lactate solution. Given the respiratory insufficiency, she was intubated and admitted to the intensive care unit (ICU). Concomitantly, she was started on hemodialysis for three hours. At presentation and after dialysis, blood samples were collected to measure concentrations of the ingested drugs. Thereafter, symptomatic treatment with sodium bicarbonate (8.4%, 100 ml) and bicarbonate tablets was continued for the persisting non-anion gap metabolic acidosis. The following day the patient could be weaned off the ventilator. Three days after she was admitted she could be discharged from the ICU without residual symptoms.

**Table 2:** drug and metabolite concentrations pre and post dialysis of venlafaxine and quetiapine following a poly drug intoxication (see Table 1 for the case report).

	Dose	Weight adjusted dose	At presentation, pre dialisis	4.5 h later after 3 h of hemodialysis
Topiramate	510 tablets of 50 mg = 25.500 mg	392,3 mg/Kg	313.5 mg/l	132.5 mg/L
Venlafaxine	170 tablets of 75 mg = 13.125 mg	201,9 mg/Kg	3509 µg/l	3109 µg/L
Desmethyl-venlafaxine			969 µg/L	1075 µg/L
Venlafaxine + Desmethyl-venlafaxine			4478 µg/L	4184 µg/L
Quetiapine	30 tablets of 25 mg = 750 mg	11,5 mg/Kg	246 µg/L	42 µg/L

### 3.1 Search results

**Fig. 2:**
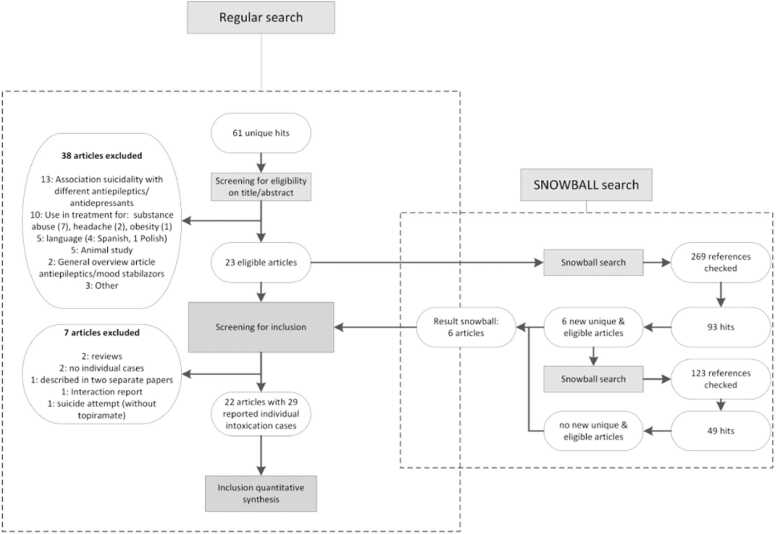
Flowchart of search, selection and review process. The used search strategy was: (topiramate [Supplementary Concept] OR Topiramate [Title/Abstract]) AND ("suicide" [MeSH Terms] OR "drug overdose" [MeSH Terms] OR intoxication)). Overview of excluded articles are provided in supplemental digital content 1.

The latest PubMed database search update was on 17 September 2021 (see Fig. 2, the flowchart of the search, selection and review process). The initial 61 unique hits were screened based on title and abstract; 23 were categorized as eligible, and a total of 38 articles were excluded because they did not match eligibility criteria for various reasons (see Fig. 2 and Supplemental Digital Content 1). The 269 references of the 23 eligible articles were snowball-searched, which yielded a total of 93 articles, among which 87 double hits and six new eligible articles remained. The references (n = 123) of these articles were also searched and screened for eligibility, which yielded 49 double hits and identified no new articles.

A total of 29 eligible articles reporting topiramate intoxications were thus identified through regular and snowball searches (23 and six articles, respectively), published between 1999 and 2017. All were reviewed for individual cases and checked for inclusion and exclusion criteria. Seven articles were ultimately excluded, two papers were reviews [10.], [11.], two did not report individual cases [18.], [19.], one case was described in two separate papers [20.], [21.], one was an interaction report and one was a suicide attempt without topiramate following initiation of topiramate [13].

Ultimately, 29 individual cases were included from the 22 included articles [21.], [22.], [23.], [24.], [25.], [26.], [27.], [28.], [29.], [30.], [31.], [32.], [33.], [34.], [35.], [36.], [37.], [38.], [39.], [40.], [41.], [42.] (see Table 3 for summarized results and Supplemental Content 2 for complete results). The majority of patients were female (n = 23/29, 79%); their age range was 2–44 years (median 21) and their weight range was 21–128 Kg (median 55 Kg, n = 17). In the large majority of cases, topiramate was regularly prescribed (18/29, 62%) or were probably on such regular treatment (3/29, 10%). For a minority, topiramate was not prescribed to the patient described in the case (n = 7/29, 24%), or this was unclear (n = 1/29, 3%). The total amount of ingested topiramate was available in 18 cases, ranging from 175 to 40,000 mg; relative to weight, the ingested amount of topiramate ranged from 4.0 to 487.8 mg/kg. The usual maximum maintenance dose in adults is 500 mg daily in two doses. Topiramate concentrations were reported in eight cases, ranging from 3.7 to 356.6 mg/L, with the reference therapeutic range being 2–30 mg/L. Most intoxications were described as poly-drug intoxications (15/28, 54%).

**Table 3:** summarized results of the cases reporting topiramate intoxications. For complete results, see Supplementary content 2.

Case ID	Patient gender	Patient age	Patient body weight (Kg)	Topiramate (ingested dose in mg)	Topiramate (ingested dose in mg//kg body weight)	Co-ingestion		Seizures	Symptoms	Concentration topiramate (Measured, ug/ml)	Gastric lavage	Activated charcoal/sorbitol	Intubation	Hemodialysis	Outcome
Sarfaraz_1[22]	F	21	43.7	1500	34.3	Yes		None	Asymptomatic	n.a.	No	Yes	No	No	recovered without sequelae
Sarfaraz_2[22]	F	21	43.7	175	4.0	Yes		None	Asymptomatic	n.a.	No	Yes	No	No	recovered without sequelae
Vazques_1[23]	F	24	n.a.	n.a.	n.a.	Yes		None	low level of consciousness (GCS 6), pale skin, and reactive mydriatic pupils.	n.a.	Yes	Yes	No	No	not specifically described
Rubio_1[24]	F	38	n.a.	n.a.	n.a.	Yes		None	tending to somnolence,	n.a.	No	Yes	No	No	recovered
Beer_1[25]	F	41	128	n.a.	n.a.	Yes		n.a.	Unresponsive	49	No	No	No	No	dead
Brandt_1[26]	M	21	108	8000	74.1	No		Non-convulsive status epilepticus	drowsy and disoriented, initially responded inappropriately, and then became unresponsive	144.6	No	No	No	No	recovered
Lynch_1[27]	F	37	n.a.	n.a.	n.a.	Yes		interpreted as seizure activity.	Unresponsive, later comatose	356.6	No	No	Yes	No	recovered
Bauer_1[28]	F	27	78	2000	25.6	Yes		generalized tonic–clonic seizures	Comatose, generalized tonic–clonic seizures,	3.7	Yes	Yes	Yes	No	complete physical recovery
Meral_1[29]	F	2	n.a.	n.a.	6	No		seizures and myoclonic activity	seizures and myoclonic activity.	n.a.	No	No	No	No	myoclonic activity had ceased
Wisniewski_1[21]	F	18	55	4000	72.7	No		None	Somnolence, vertigo, mydriasis without reaction to light	n.a.	No	No	No	No	recovered without sequelae
Wisniewski_2[21]	F	16	55	12000	218.2	No		None	Agitation, confusion, mydriasis with slow reaction to light	n.a.	No	No	No	No	recovered without sequelae
Wisniewski_3[21]	F	19	55	1500	27.3	No		None	Somnolence	n.a.	Yes	No	No	No	recovered without sequelae
Wisniewski_4[21]	M	38	80	2500	31.3	Yes		Three secondarily generalized seizures	Coma, somnolence, vertigo, bradykinesia, and bradyphasia	n.a.	No	No	No	No	recovered without sequelae
Wisniewski_5[21]	M	19	70	750	10.7	No		None	Asymptomatic	n.a.	Yes	No	No	No	recovered without sequelae
Wisniewski_6[21]	F	16	52	750	14.4	No		None	Somnolence	n.a.	No	No	No	No	recovered without sequelae
Kaufman_1[30]	F	23	n.a.	n.a.	n.a.	Yes		None	Somnolent, diaphoretic, tachycardic, muscle rigidity	n.a.	No	No	Yes	No	discharged, resolution of hyperthermia and decreasing CPK levels
Lin_1[31]	F	2.8	21	n.a.	n.a.	Yes		None	Ataxia (could not walk, regressed to crawling at home), Slurred speech, visual hallucinations	n.a.	No	No	No	No	resolution of symptoms
Brar_1[32]	M	13	n.a.	n.a.	n.a.	No		None	acute confusion, agitation, incoherent speech, visual hallucinations, memory deficits, decreased attention, dyscalculia, and cognitive slowing	n.a.	No	No	No	No	almost complete resolution of symptoms (36 hr.)
Anand_1[33]	F	15	n.a.	450	n.a.	No		None	After about 2–3 h bradykinesia and bradyphasia	n.a.	Yes	No	No	No	resolution of symptoms
Chung_1[34]	F	17	50	800	16	No		None	somnolent and unintelligible speech; later cycle between periods of calmness and combativeness	n.a.	No	Yes	No	No	resolution of symptoms (24 hr.)
Langman_1[35]	F	44	n.a.	n.a.	n.a.	Yes		None	dead	170	No	No	No	No	dead
Colom_1[36]	F	30	62	450	7.3	No		None	decreased cognition, dulled thinking, blunted mental reactions, blurred vision, paresthesia, moderate sleepiness, and GI disturbances	n.a.	No	No	No	No	resolution of symptoms (rapidly)
Dhelens_1[37]	M	10	35	700	20	No		None	Somnolence, agitation, hallucinations	n.a.	No	No	No	No	recovered.
Traub_1[38]	F	5	n.a.	n.a.	n.a.	No		None	Sleepiness, couldn’t feel anything, arching movements of back. Perdivation and repetitive mouthing movements	10.5	No	No	No	No	complete resolution of symptoms in 24 h
Smith_1[39]	F	24	n.a.	4000	n.a.	No		None	asymptomatic	18	Yes	No	No	No	recovered (did not develop any adverse sequelae)
Fakhoury_1[40]	F	42	50	20000	400	Yes		intermittent generalized tonic clonic activity	Unresponsive, hypotensive, intermittent seizures	n.a.	Yes	Yes	Yes	No	resolution of symptoms (in 12 hr), non-anion-gap metabolic acidosis persisted for > 6 days before resolving
Fakhoury_2[40]	M	36	82	40000	487	No		convulsive status epilepticus	A first asymptomatic, 2 h later convulsive status epilepticus	n.a.	No	No	Yes	No	remained somnolent until Day 5. Persistent nonanion-gap metabolic acidosis and alkaline urine did not resolve until Day 7
Kemmerer_1[42]	F	29	n.a.	3000	n.a.	Yes		None	initially hypertension and tachycardia, but quickly resolved. Decreased consciousness	n.a.	No	Yes	No	No	resolution of symptoms after 3 days
Mozayani_1[41]	F	15	n.a.	n.a.	n.a.		n.a.	None	Unresponsive	8.9	No	No	No	No	dead

The clinical features of the topiramate intoxications were various and were heavily influenced by other drugs used. Somnolence and unresponsiveness were the most common features in most cases. Seizures were also reported (8/28, 29%). Laboratory analysis revealed non-anion gap metabolic acidosis (low bicarbonate and elevated chloride) or not-further-specified acidosis in the large majority of cases where blood gas analysis was reported (acidosis 11/15 or 73%; specific non-anion gap metabolic acidosis 6/15 or 40%).

The initiated therapy in most cases was conservative management, including supportive care and general anti-intoxication measures. Gastrointestinal decontamination with, for example, activated charcoal and sorbitol was used predominantly (8/29; 28%), but gastric lavage was prescribed in almost a quarter of the cases (7/29, 24%). Intubation was sometimes needed (5/29, 17%) either as a consequence of somnolence caused by topiramate, because of other drugs in the case of poly-drug intoxication or because of benzodiazepine treatment for seizures induced by the topiramate intoxication. In none of the cases was hemodialysis used.

The described outcomes of topiramate intoxications included in the case reports was quite limited with regard to eventual follow-up. In the described cases, three patients died, with an overall mortality of 10% (n = 3/29). Most patients were discharged with resolution of all symptoms, sometimes with a longer-persisting non-anion gap metabolic acidosis.

## 4 Discussion

Serious topiramate intoxications can result in seizures, hemodynamic instability, coma, severe metabolic acidosis and even death, although combined intoxications were common. Therefore, treatment is based on symptoms and most frequently is supportive. Literature on blood concentrations of topiramate following (auto)intoxications is limited. Invasive treatment such as intubation and hemodialysis is used infrequently. If symptoms are refractory to symptomatic treatment, such as our case demonstrated, hemodialysis can reduce topiramate concentrations and symptomatology.

Compared to the reviewed literature, this case report provides the first pharmacokinetic data following hemodialysis after a massive topiramate overdose, with, in this case, co-ingestion of venlafaxine and quetiapine. Hemodialysis was not used in any of the included cases. Previously, Wade et al. mention that hemodialysis is effective at removing this drug and that it could be reserved for severe symptoms such as seizures, refractory hypotension or significant metabolic acidosis [10]. Nevertheless, this seems to be an expert opinion without reference to literature, based on the pharmacokinetic properties of topiramate. The pharmacokinetics of topiramate include a low molecular weight and, therefore, size (molecular weight 339.4 Da), a volume distribution of 0.6–0.8 L/kg, very low protein binding (9–17%) and primarily renal excretion (~60%)[43].

The clearance of topiramate in therapeutic doses has been described in literature, with a reported linear clearance in the dose range of 100–800 mg [44]. In a case report by Brandt in 2010, this linear (zero-order) pharmacokinetics is confirmed in topiramate concentrations above 50 ug/ml [26]. In this case report, we measured a reduction of topiramate concentration from 313.5 to 132.5 mg/L in 4.5 h, of which three hours involved treatment with dialysis. Pharmacokinetic modelling was omitted, given the limited measured concentrations available.

For normal or sub-therapeutic topiramate concentrations, pharmacokinetic data of dialysis do exist. In a recent literature review on anticonvulsant removal by renal replacement therapy, only one case report was included. The review concluded there was insufficient data; however, topiramate is likely removed by continuous renal replacement therapy (CRRT) [45]. In the specific case report, the observed trough concentrations were 35% lower than expected in patients with normal kidney function, demonstrating plausible removal of topiramate by continuous venovenous hemodiafiltration (CVVHDF) [46]. Recently, non-included data in the referenced review demonstrated that a single dose of topiramate (100 mg) was effectively cleared by hemodialysis in end-stage renal disease hemodialysis patients [17]. During a three-hour hemodialysis treatment, plasma topiramate concentrations dropped by approximately 50% [17]. This seems comparable with the limited data in our case. Besides full hemodialysis, Smetana et al. describe the possible effectiveness of continuous venovenous hemodialysis (CVVHD) in a 59-year-old man with refractory status epilepticus while receiving topiramate (200 mg BID). After three days, sub-therapeutic trough concentrations were measured, most likely due to CVVHD [16]. Altogether, the advice to start hemodialysis in topiramate intoxications in the summary of product characteristics of topiramate seems to be based on the effect of renal replacement therapy in low therapeutic doses of topiramate, and not yet on evidence or experience with dialysis in supra-therapeutic doses due to intoxications, although an extrapolation of the observed effectiveness in higher concentrations of topiramate is logical and theoretically sound.

Next to the measured topiramate concentrations following hemodialysis, we also demonstrated the inability of hemodialysis to remove venlafaxine, including desmethyl-venlafaxine, and the remarkable reduction in the serum quetiapine concentration following hemodialysis. For quetiapine, the reduction in the serum concentration could be transient, and a rebound phenomenon could be expected due to redistribution given quetiapine’s large volume of distribution.

A major limitation of the review is the selection bias of reported cases. Outstanding cases with possible severe symptoms or outcomes would be more likely to be reported, and “common” intoxications could be reported less often. Moreover, in our case report, we measured topiramate concentrations only once directly after dialysis, which makes pharmacokinetic modeling unreliable. A strength of this report is the summary of the present knowledge regarding topiramate intoxications included in case reports. Moreover, we provide the first case report with topiramate for which hemodialysis was initiated and pre- and post-dialysis concentrations were measured and reported.

## 5 Conclusion

Topiramate is a commonly used drug in the treatment of a wide range neurologic conditions. In our review we demonstrated the common practice of treating auto-intoxications with supportive measures. Invasive treatments such as hemodialysis are infrequently used, and toxicokinetic data are very limited. In this report we describe a patient with a massive intoxication that resulted in coma and seizures. Given the high doses of ingested topiramate, hemodialysis was initiated, which considerably enhanced the clearance of both topiramate and quetiapine.

## Ethical approval

This article does not contain any studies with animals performed by any of the authors.

## Informed consent

The review of previous cases described encompasses a secondary analysis of these described cases and therefore no separate consent procedures were deemed necessary or applicable. For the described case, identifying details were omitted where possible, to safeguard confidentiality. Informed consent was obtained from the patient.

## Declaration of Competing Interest

The authors have no conflicts of interest.

## Data Availability

No data was used for the research described in the article.
