# Supplementary material for: Topiramate intoxications & hemodialysis – Literature review and the first case report of a massive suicidal intoxication treated with hemodialysis
Source: Toxicol Rep. 2022 Aug 12;9:1639–46. doi: 10.1016/j.toxrep.2022.08.004 (PMC9764168; doi:10.1016/j.toxrep.2022.08.004)
Supplement: Supplementary file 1 — Supplementary material [file mmc1.pdf]

Supplemental digital content 1 - Overview of excluded articles

Toxicology reports

Topiramate intoxications & hemodialysis – Literature review and the first case report of a massive suicidal intoxication treated with hemodialysis

Tim Schutte[1] , Anne van Tellingen[2], Janneke van den Broek[2], Marloes ten Brink[2], Marleen G. van Agtmael-Boerrigter[2]

[1] Amsterdam UMC location Vrije Universiteit Amsterdam, Department of Internal Medicine & Department of Medical Oncology, Boelelaan 1117, Amsterdam, The Netherlands

[2] Zaans Medisch Centrum, Zaandam, the Netherlands

The screening for eligibility on title/abstract resulted in a total of 38 articles that were excluded

**Reason for exclusion**		**References**
Association suicidality with different antiepileptics/antidepressants including topiramate (n=13)		(1-13)
Use in treatment (‘new indication’) (n=10)	for substance abuse (n=7)	(14-20)
	headache (n=2)	(21, 22)
	obesity (n=1)	(23)
Language (n=5)	Spanish (n=4	(24-27)
	Polish (n=1)	(28)
Animal study (n=5)		(29-33)
General overview article antiepileptics/mood stabilazors (n=2)		(34, 35)
Other (n=3)	Improvement in neuropsychological functions after withdrawal of topiramate	(36)
	Carbamazepine intoxication due to clobazam-carbamazepine interaction	(37)
	Detection of drugs in alcohol-positive blood samples of drivers	(38)

At final assessment, a total of 7 articles were excluded

**Reason for exclusion**	**References**
Reviews (n=2)	(39, 40)
No individual (intox-)cases described (n=2)	(41, 42)
Case described in two separate papers (n=1)	(43, 44)*
Interaction report (n=1)	(45)
Suicide attempt (without topiramate) (n=1)	(46)

* n=1 exclued, references for both papers are provided.

References:

1. Schuerch M, Gasse C, Robinson NJ, Alvarez Y, Walls R, Mors O, et al. Impact of varying outcomes and definitions of suicidality on the associations of antiepileptic drugs and suicidality: comparisons from UK Clinical Practice Research Datalink (CPRD) and Danish national registries (DNR). Pharmacoepidemiol Drug Saf. 2016;25 Suppl 1:142-55.
2. Fountoulakis KN, Gonda X, Baghai TC, Baldwin DS, Bauer M, Blier P, et al. Report of the WPA section of pharmacopsychiatry on the relationship of antiepileptic drugs with suicidality in epilepsy. Int J Psychiatry Clin Pract. 2015;19(3):158-67.
3. Siamouli M, Samara M, Fountoulakis KN. Is antiepileptic-induced suicidality a data-based class effect or an exaggeration? A comment on the literature. Harv Rev Psychiatry. 2014;22(6):379-81.
4. Pugh MJ, Copeland LA, Zeber JE, Wang CP, Amuan ME, Mortensen EM, et al. Antiepileptic drug monotherapy exposure and suicide-related behavior in older veterans. J Am Geriatr Soc. 2012;60(11):2042-7.
5. Fountoulakis KN, Gonda X, Samara M, Siapera M, Karavelas V, Ristic DI, et al. Antiepileptic drugs and suicidality. J Psychopharmacol. 2012;26(11):1401-7.
6. Kanner AM. Are antiepileptic drugs used in the treatment of migraine associated with an increased risk of suicidality? Curr Pain Headache Rep. 2011;15(3):164-9.
7. Machado RA, Espinosa AG, Melendrez D, Gonzalez YR, Garcia VF, Rodriguez YQ. Suicidal risk and suicide attempts in people treated with antiepileptic drugs for epilepsy. Seizure. 2011;20(4):280-4.
8. Ziemba KS, O'Carroll CB, Drazkowski JF, Wingerchuk DM, Hoffman-Snyder C, Wellik KE, et al. Do antiepileptic drugs increase the risk of suicidality in adult patients with epilepsy?: a critically appraised topic. Neurologist. 2010;16(5):325-8.
9. Andersohn F, Schade R, Willich SN, Garbe E. Use of antiepileptic drugs in epilepsy and the risk of self-harm or suicidal behavior. Neurology. 2010;75(4):335-40.
10. Patorno E, Bohn RL, Wahl PM, Avorn J, Patrick AR, Liu J, et al. Anticonvulsant medications and the risk of suicide, attempted suicide, or violent death. JAMA. 2010;303(14):1401-9.
11. Kalinin VV. Suicidality and antiepileptic drugs: is there a link? Drug Saf. 2007;30(2):123-42.
12. Born C, Dittmann S, Post RM, Grunze H. Newer prophylactic agents for bipolar disorder and their influence on suicidality. Arch Suicide Res. 2005;9(3):301-6.
13. Abraham G. Topiramate-induced suicidality. Can J Psychiatry. 2003;48(2):127-8.
14. Crist RC, Doyle GA, Kampman KM, Berrettini WH. A delta-opioid receptor genetic variant is associated with abstinence prior to and during cocaine dependence treatment. Drug Alcohol Depend. 2016;166:268-71.
15. Kranzler HR, Wetherill R, Feinn R, Pond T, Gelernter J, Covault J. Posttreatment effects of topiramate treatment for heavy drinking. Alcohol Clin Exp Res. 2014;38(12):3017-23.
16. Kranzler HR, Armeli S, Tennen H, Gelernter J, Covault J. GRIK1 genotype and daily expectations of alcohol's positive effects moderate the reduction of heavy drinking by topiramate. Exp Clin Psychopharmacol. 2014;22(6):494-501.
17. Liang J, Olsen RW. Alcohol use disorders and current pharmacological therapies: the role of GABA(A) receptors. Acta Pharmacol Sin. 2014;35(8):981-93.
18. Likhitsathian S, Saengcharnchai P, Uttawichai K, Yingwiwattanapong J, Wittayanookulluk A, Srisurapanont M. Cognitive changes in topiramate-treated patients with alcoholism: a 12-week prospective study in patients recently detoxified. Psychiatry Clin Neurosci. 2012;66(3):235-41.
19. Walter M, Wiesbeck GA. [Pharmacotherapy of substance dependence and withdrawal syndromes]. Ther Umsch. 2009;66(6):449-57.
20. Hughes JR. Alcohol withdrawal seizures. Epilepsy Behav. 2009;15(2):92-7.
21. Ye L, Karlapati SK, Lippmann S. Topiramate for post-electroconvulsive therapy headaches. J ECT. 2013;29(3):e49.
22. Lanteri-Minet M, Demarquay G, Alchaar H, Bonnin J, Cornet P, Douay X, et al. [Management of chronic daily headache in migraine patients: medication overuse headache and chronic migraine. French guidelines (French Headache Society, French Private Neurologists Association, French Pain Society)]. Rev Neurol (Paris). 2014;170(3):162-76.
23. Wilding J, Van Gaal L, Rissanen A, Vercruysse F, Fitchet M, Group O-S. A randomized double-blind placebo-controlled study of the long-term efficacy and safety of topiramate in the treatment of obese subjects. Int J Obes Relat Metab Disord. 2004;28(11):1399-410.

24. Gracia-Naya M, Hernando-Quintana N, Latorre-Jimenez AM, Rios C, Artal-Roy J, Garcia-Gomara MJ, et al. [Does medication abuse in patients with chronic migraine influence the effectiveness of preventive treatment with topiramate?]. Rev Neurol. 2014;59(9):385-91.
25. Hidalgo SF, Prieto de Paula JM. [Acute topiramate intoxication: cause of metabolic acidosis and central hyperventilation]. Med Clin (Barc). 2011;136(5):224-5.
26. Barcelo B, Yates C, Castanyer B, Puiguriguer J. [Delayed seizures after topiramate, venlafaxine and quetiapine overdose]. Med Clin (Barc). 2010;135(4):191-2.
27. Garcia-Gil D, Perez Vazquez V, Asencio Mendez C, Garcia-Torrejon J. [Acute topiraranate toxicity to suicidal attempt]. Med Clin (Barc). 2009;133(19):766-7.
28. Lukasik K, Piatkowska K, Pietrzak B. [The role of the glutamatergic system in the pathogenesis and treatment of alcohol dependence]. Postepy Hig Med Dosw (Online). 2010;64:534-43.
29. Yamamura S, Hamaguchi T, Ohoyama K, Sugiura Y, Suzuki D, Kanehara S, et al. Topiramate and zonisamide prevent paradoxical intoxication induced by carbamazepine and phenytoin. Epilepsy Res. 2009;84(2-3):172-86.
30. Chen YC, Holmes A. Effects of topiramate and other anti-glutamatergic drugs on the acute intoxicating actions of ethanol in mice: modulation by genetic strain and stress. Neuropsychopharmacology. 2009;34(6):1454-66.
31. Lagrue E, Chalon S, Bodard S, Saliba E, Gressens P, Castelnau P. Lamotrigine is neuroprotective in the energy deficiency model of MPTP intoxicated mice. Pediatr Res. 2007;62(1):14-9.
32. Harrison PK, Sheridan RD, Green AC, Tattersall JE. Effects of anticonvulsants on soman-induced epileptiform activity in the guinea-pig in vitro hippocampus. Eur J Pharmacol. 2005;518(2-3):123-32.
33. Sharma HS, Muresanu DF, Patnaik R, Stan AD, Vacaras V, Perju-Dumbrav L, et al. Superior neuroprotective effects of cerebrolysin in heat stroke following chronic intoxication of Cu or Ag engineered nanoparticles. A comparative study with other neuroprotective agents using biochemical and morphological approaches in the rat. J Nanosci Nanotechnol. 2011;11(9):7549-69.
34. Ferrier IN. Developments in mood stabilisers. Br Med Bull. 2001;57:179-92.
35. Yoon Y, Jagoda A. New antiepileptic drugs and preparations. Emerg Med Clin North Am. 2000;18(4):755-65.
36. Kockelmann E, Elger CE, Helmstaedter C. Significant improvement in frontal lobe associated neuropsychological functions after withdrawal of topiramate in epilepsy patients. Epilepsy Res. 2003;54(2-3):171-8.
37. Genton P, Nguyen VH, Mesdjian E. Carbamazepine intoxication with negative myoclonus after the addition of clobazam. Epilepsia. 1998;39(10):1115-8.
38. Kim E, Choe S, Lee J, Jang M, Choi H, Chung H. Detection of drugs in 275 alcohol-positive blood samples of Korean drivers. Forensic Sci Int. 2016;265:186-92.
39. Wade JF, Dang CV, Nelson L, Wasserberger J. Emergent complications of the newer anticonvulsants. J Emerg Med. 2010;38(2):231-7.
40. Lofton AL, Klein-Schwartz W. Evaluation of toxicity of topiramate exposures reported to poison centers. Hum Exp Toxicol. 2005;24(11):591-5.
41. Wills B, Reynolds P, Chu E, Murphy C, Cumpston K, Stromberg P, et al. Clinical outcomes in newer anticonvulsant overdose: a poison center observational study. J Med Toxicol. 2014;10(3):254-60.
42. Gordon AM, Logan BK. Topiramate-positive death-investigation and impaired-driving cases in Washington State. J Anal Toxicol. 2006;30(8):599-602.
43. Anand JS, Chodorowski Z, Wisniewski M. Seizures induced by topiramate overdose. Clin Toxicol (Phila). 2007;45(2):197.
44. Wisniewski M, Lukasik-Glebocka M, Anand JS. Acute topiramate overdose--clinical manifestations. Clin Toxicol (Phila). 2009;47(4):317-20.
45. Mack CJ, Kuc S, Mulcrone SA, Pilley A, Grunewald RA. Interaction of topiramate with carbamazepine: two case reports and a review of clinical experience. Seizure. 2002;11(7):464-7.
46. Christman DS, Faubion MD. Suicide attempt following initiation of topiramate. Am J Psychiatry. 2007;164(4):682-3.
