# Supplementary material for: Topiramate intoxications & hemodialysis – Literature review and the first case report of a massive suicidal intoxication treated with hemodialysis
Source: Toxicol Rep. 2022 Aug 12;9:1639–46. doi: 10.1016/j.toxrep.2022.08.004 (PMC9764168; doi:10.1016/j.toxrep.2022.08.004)
Supplement: Supplementary file 2 — Supplementary material [file mmc2.pdf]

Supplemental digital content 2 – extended case characteristics of case reports on topiramate intoxications

Toxicology reports

Topiramate intoxications & hemodialysis – Literature review and the first case report of a massive suicidal intoxication treated with hemodialysis

Tim Schutte[1] , Anne van Tellingen[2], Janneke van den Broek[2], Marloes ten Brink[2], Marleen G. van Agtmael-Boerrigter[2]

[1] Amsterdam UMC location Vrije Universiteit Amsterdam, Department of Internal Medicine & Department of Medical Oncology, Boelelaan 1117, Amsterdam, The Netherlands

[2] Zaans Medisch Centrum, Zaandam, the Netherlands

Titel	Case ID	Patient sexe	Patient age	Patient body weight (kg)	Topiramate (ingested dose in mg)	Topiramate (ingested dosein mg/Kg body weigt)	Co-ingestion	Seizures?	Symptoms	Concentration Topiramate (Measured, ug/ml)	Bloodgas analysis	Treatment	Gastric lavage	Activated charcoal/sorbitol	Intubation	Hemodialysis	Outcome
Levetiracetam and topiramate poisoning: Two overdoses on those drugs with no lasting effects (1)	Sarfaraz_2017_1	F	21	43.7	1500	34.3	levetiracetam	None	Asymptomatic	n.a.	n.a.	activated charcoal	No	Yes	No	no	recovered without sequelae
	Sarfaraz_2017_2	F	21	43.7	175	4.0	levetiracetam	None	Asymptomatic	n.a.	no abnormalities	activated charcoal	No	Yes	No	no	recovered without sequelae
Coma Blisters after an Overdose of Central Nervous System Depressants (2)	Vazques_2017_1	F	24	n.a.	n.a.	n.a.	duloxetine, quetiapine, clorazepate	None	low level of consciousness (GCS 6), pale skin, and reactive mydriatic pupils.	n.a.	n.a.	oxygen, fluid therapy, gastric lavage, and activated charcoal.	Yes	Yes	No	no	not specifically described
Metabolic acidosis and topiramate. Use of Winters' formula (3)	Rubio_2014_1	F	38	n.a.	n.a.	n.a.	Lorazepam	None	tending to somnolence,	n.a.	metabolic acidosis with normal anion gap	activated charcoal.	No	Yes	No	no	recovered
A fatal intoxication case involving topiramate (4)	Beer_2010_1	F	41	128	n.a.	n.a.	citalopram and flunitrazepam	n.a.	Unresponsive	49		Continued resuscitation efforts	No	No	No	no	Dead
Topiramate overdose: a case report of a patient with extremely high topiramate serum concentrations and nonconvulsive status epilepticus (5)	Brandt_2010_1	M	21	108	8000	74.1	no	Non-convulsive status epilepticus	drowsy and disoriented, initially responded inappropriately, and then became unresponsive	144.6		no	No	No	No	no	recovered
Clinical effects and toxicokinetic evaluation following massive topiramate ingestion. (6)	Lynch_2010_1	F	37	n.a.	n.a.	n.a.	ibuprofen	interpreted as seizure activity.	Unresponsive, later comatose	356.6	nonanion gap metabolic acidosis (pH 7.26, pCO2 41 mmHg, and pO2 320 mmHg, bicarbonate 21 mEq/L anion gap 16.	3.5 mg i.v. lorazepam (seizures); naloxone	No	No	Yes	no	Recovered
Lacosamide intoxication in attempted suicide (7)	Bauer_2010_1	F	27	78	2000	25.6	lacosamide, gabapentin, zonisamide	generalized tonic–clonic seizures	Comatose, generalized tonic–clonic seizures,	3.7	mild acidosis (pH 7.296)	sodium sulfate and carbon	Yes	Yes	Yes	no	complete physical recovery
Simple dosage inaccuracy might be the cause of serious side effects of topiramate (8)	Meral_2009_1	F	2	n.a.	n.a.	6	no	seizures and myoclonic activity	seizures and myoclonic activity.	n.a.	n.a.	dose reduction	No	No	No	no	myoclonic activity had ceased
Acute topiramate overdose--clinical manifestations (9)	Wisniewski_2009_1	F	18	55	4000	72.7	no	None	Somnolence, vertigo, mydriasis without reaction to light	n.a.	Metabolic acidosis with pH 7.28; HCO3 16.6 mEql/l; BE – 9,1; pCO2 – 30 mmHg – lasted for 3 days	Supportive, bicarbonate 80 mEq/d for 4 days	No	No	No	no	recovered without sequelae
	Wisniewski_2009_2	F	16	55	12000	218.2	no	None	Agitation, confusion, mydriasis with slow reaction to light	n.a.	Metabolic acidosis with pH 7.33, HCO3 15.3 mEq/l; BE – 8.9; pCO2 – 29, 1 mmHg – lasted for 7 days	Bicarbonate, day 1 – 210 mEq/d, day 2 – 120 mEq/d, days 3 – 7 – 80 mEq/d	No	No	No	no	recovered without sequelae
	Wisniewski_2009_3	F	19	55	1500	27.3	no	None	Somnolence	n.a.	Without any biochemical abnormalities (including acidosis)	Supportive	Yes	No	No	no	recovered without sequelae
	Wisniewski_2009_4	M	38	80	2500	31.3	Ethanol	Three secondarily generalized seizures	Coma, somnolence, vertigo, bradykinesia, and bradyphasia	n.a.	Metabolic acidosis pH 7.34; HCO3 17.3 mEq/l; BE – 7.5; pCO2 32; mmHg – lasted for 3 days	Supportive, diazepam, clonazepam, bicarbonate, day 1 – 60 mEq/d	No	No	No	no	recovered without sequelae
	Wisniewski_2009_5	M	19	70	750	10.7	no	None	Asymptomatic	n.a.	Without any biochemical abnormalities (including acidosis)	Supportive	Yes	No	No	no	recovered without sequelae
	Wisniewski_2009_6	F	16	52	750	14.4	no	None	Somnolence	n.a.	Compensated metabolic acidosis with pH 7.44; HCO3 16.8 mEq/l; BE – 4,2 pCO2 22 mmHg – lasted for 3 days	Supportive, bicarbonate; day 1 – 60 mEq/d, day 2 – 40 mEq/d	No	No	No	no	recovered without sequelae
Neuroleptic malignant syndrome and serotonin syndrome in the critical care setting: case analysis (10)	Kaufman_2006_1	F	23	n.a.	n.a.	n.a.	Mixed polydrug intox venlafaxine, topiramate, divalproex sodium,	None	Somnolent, diaphoretic, tachycardic, muscle rigidity	n.a.	n.a.	Intubation, lorazepam 0.5 to 1.0 mg/hr	No	No	Yes	no	discharged, resolution of hyperthermia and decreasing CPK levels

							risperidone and carbamazepine.										
Pediatric case report of topiramate toxicity (11)	Lin_2006_1	F	2.8	21	n.a.	n.a.	acetaminophen, dextromethorphan, pseudophedrine, doxylamine	None	Ataxia (could not walk, regressed to crawling at home), Slurred speech, visual hallucinations	n.a.	n.a.	supportive.	No	No	No	no	resolution of symptoms
Acute mental status changes with topiramate (12)	Brar_2005_1	M	13	n.a.	n.a.	n.a.	no	None	acute confusion, agitation, incoherent speech, visual hallucinations, memory deficits, decreased attention, dyscalculia, and cognitive slowing	n.a.	n.a.	2,5 mg haloperidol	No	No	No	no	almost complete resolution of symptoms (36 hr.)
Acute suicidal intoxication with topiramate (13)	Anand_2005_1	F	15	n.a.	450	n.a.	no	None	After about 2-3 hours bradykinesia and bradyphasia	n.a.	n.a.	supportive treatment (gastric lavage)	Yes	No	No	no	resolution of symptoms
Intentional topiramate ingestion in an adolescent female (14)	Chung_2004_1	F	17	50	800	16	no	None	somnolent and unintelligible speech; later cycle between periods of calmness and combativeness	n.a.	2 hours after ingestion, mild metabolic acidosis: pH 7.38, PCO2 32.4 mm Hg, PO2 108 mm Hg, and HCO3 19.5 mEq/L, base excess – 4.1 mEq/L, serum bicarbonate 18 mEq/L, anion gap 13	activated charcoal 50 g with sorbital via a nasogastric tube.	No	Yes	No	no	resolution of symptoms (24 hr.)
Fatal acute topiramate toxicity (15)	Langman_2003_1	F	44	n.a.	n.a.	n.a.	Ethanol	None	dead	170	n.a.	none	No	No	No	no	dead
Topiramate abuse in a bipolar patient with an eating disorder (16)	Colom_2001_1	F	30	62	450	7.3	no	None	decreased cognition, dulled thinking,<br>blunted mental reactions, blurred vision, paresthesia, moderate sleepiness, and GI disturbances	n.a.	n.a.	dose reduction	No	No	No	no	resolution of symptoms (rapidly)
Intoxication volontaire pédiatrique au topiramate : à propos d'un cas / One Case of Child Topiramate Self-Poisoning (17)	Dhelens_2011_1	M	10	35	700	20	no	None	Somnolence, agitation, hallucinations	n.a.	non-anion gap metabolic acidosis Na 136 HCO3 16 Cl 109	supportive, rehydration	No	No	No	no	recovered.
Acute topiramate toxicity (18)	Traub_2003_1	F	5	n.a.	n.a.	n.a.	no	None	Sleepiness, couldn't feel anything, arching movements of back. Perdivation and repetitive mouthing movements	10.5	n.a	no	No	No	No	no	complete resolution of symptoms in 24 h
Topiramate overdose: a case report and literature review (19)	Smith_2002_1	F	24	n.a.	4000	n.a.	no	None	asymptomatic	18	no acidosis	nasogastric lavage, sorbitol, no charcoal.	Yes	No	No	no	Recovered (did not develop any adverse sequelae)
Topiramate overdose: clinical and laboratory features (20)	Fakhoury_2002_1	F	42	50	20000	400	naproxen	intermittent generalized tonic–clonic activity	Unresponsive, hypotensive, intermittent seizures	n.a.	non-anion gap metabolic acidosis	Endotracheal intubation, Gastric lavage, 50 g activated charcoal in sorbitol, benzodiazepines	Yes	Yes	Yes	no	Within 12 hours resolution of symptoms, non-anion-gap metabolic acidosis persisted for 6 more days before resolving spontaneously
	Fakhoury_2002_2	M	36	82	40000	487	no	convulsive status epilepticus	A first asymptomatic, 2 hours later convulsive status epilepticus	n.a.	Non-anion gap metabolic acidosis	Intubation, Syrup of ipecac (30 cc), benzodiazepines	No	No	Yes	no	remained somnolent until Day 5. A persistent nonanion-gap metabolic acidosis was noted in the presence of alkaline urine and did not resolve until Day 7
Non-anion gap metabolic acidosis associated with acute or chronic topiramate overdose (21)	Kemmerer_2002_1	F	29	n.a.	3000	n.a.		None	initially hypertension and tachycardia, but quickly resolved. Decreased consciousness	n.a.	non-anion gap metabolic acidosis	charcoal with sorbitol	No	Yes	No	no	resolution of symptoms after 3 days
Distribution of topiramate in a medical examiner's case (22)	Mozayani_1999_1	F	15	n.a.	n.a.	n.a.	n.a.	None	Unresponsive	8.9	n.a.	n.a.	No	No	No	no	Dead

References

1. Sarfaraz M, Syeda RH. Levetiracetam and topiramate poisoning: Two overdoses on those drugs with no lasting effects. Drug Discov Ther. 2017;11(2):115-7.
2. Vazquez-Osorio I, Gonzalvo-Rodriguez P, Rodriguez-Diaz E. Coma Blisters after an Overdose of Central Nervous System Depressants. Actas Dermosifiliogr. 2017;108(1):81-3.
3. Rubio P, Supervia A, Aguirre A, Echarte JL. Metabolic acidosis and topiramate. Use of Winters' formula. Rev Psiquiatr Salud Ment. 2014;7(2):96.
4. Beer B, Libiseller K, Oberacher H, Pavlic M. A fatal intoxication case involving topiramate. Forensic Sci Int. 2010;202(1-3):e9-11.
5. Brandt C, Elsner H, Furatsch N, Hoppe M, Nieder E, Rambeck B, et al. Topiramate overdose: a case report of a patient with extremely high topiramate serum concentrations and nonconvulsive status epilepticus. Epilepsia. 2010;51(6):1090-3.
6. Lynch MJ, Pizon AF, Siam MG, Krasowski MD. Clinical effects and toxicokinetic evaluation following massive topiramate ingestion. J Med Toxicol. 2010;6(2):135-8.
7. Bauer S, David Rudd G, Mylius V, Hamer HM, Rosenow F. Lacosamide intoxication in attempted suicide. Epilepsy Behav. 2010;17(4):549-51.
8. Meral C, Aydinoz S. Simple dosage inaccuracy might be the cause of serious side effects of topiramate. Clin Toxicol (Phila). 2009;47(7):691.
9. Wisniewski M, Lukasik-Glebocka M, Anand JS. Acute topiramate overdose--clinical manifestations. Clin Toxicol (Phila). 2009;47(4):317-20.
10. Kaufman KR, Levitt MJ, Schiltz JF, Sunderram J. Neuroleptic malignant syndrome and serotonin syndrome in the critical care setting: case analysis. Ann Clin Psychiatry. 2006;18(3):201-4.
11. Lin G, Lawrence R. Pediatric case report of topiramate toxicity. Clin Toxicol (Phila). 2006;44(1):67-9.
12. Brar B, Glazer JP, Franco K, Edwards T. Acute mental status changes with topiramate. J Am Acad Child Adolesc Psychiatry. 2005;44(8):725.
13. Sein Anand J, Chodorowski Z, Zbikowska-Bojko M. Acute suicidal intoxication with topiramate. Przegl Lek. 2005;62(6):519.
14. Chung AM, Reed MD. Intentional topiramate ingestion in an adolescent female. Ann Pharmacother. 2004;38(9):1439-42.
15. Langman LJ, Kaliciak HA, Boone SA. Fatal acute topiramate toxicity. J Anal Toxicol. 2003;27(5):323-4.
16. Colom F, Vieta E, Benabarre A, Martinez-Aran A, Reinares M, Corbella B, et al. Topiramate abuse in a bipolar patient with an eating disorder. J Clin Psychiatry. 2001;62(6):475-6.
17. Dhelens C, Tichadou L, Glaizal M, Hayek MY, De Haro L. [One case of child topiramate self-poisoning]. Therapie. 2011;66(3):295-6.
18. Traub SJ, Howland MA, Hoffman RS, Nelson LS. Acute topiramate toxicity. J Toxicol Clin Toxicol. 2003;41(7):987-90.
19. Smith AG, Brauer HR, Catalano G, Catalano MC. Topiramate Overdose: A Case Report and Literature Review. Epilepsy Behav. 2001;2(6):603-7.
20. Fakhoury T, Murray L, Seger D, McLean M, Abou-Khalil B. Topiramate Overdose: Clinical and Laboratory Features. Epilepsy Behav. 2002;3(2):185-9.
21. Kemmerer D SK, Tomassoni A. Non-anion gap metabolic acidosis associated with acute on chronic topiramate overdose. J Toxicol Clin Toxicol. 2002;40.
22. Mozayani A, Carter J, Nix R. Distribution of topiramate in a medical examiner's case. J Anal Toxicol. 1999;23(6):556-8.
